# Associations between Weather, Air Quality and Moderate Extreme Cancer-Related Mortality Events in Augsburg, Southern Germany

**DOI:** 10.3390/ijerph182211737

**Published:** 2021-11-09

**Authors:** Patrick Olschewski, Irena Kaspar-Ott, Stephanie Koller, Gerhard Schenkirsch, Martin Trepel, Elke Hertig

**Affiliations:** 1 Faculty of Medicine, University of Augsburg, 86159 Augsburg, Germany; irena.kaspar-ott@med.uni-augsburg.de (I.K.-O.); stephanie.koller@med.uni-augsburg.de (S.K.); martin.trepel@uk-augsburg.de (M.T.); elke.hertig@med.uni-augsburg.de (E.H.); 2 Comprehensive Cancer Center, Augsburg University Medical Center, 86156 Augsburg, Germany; gerhard.schenkirsch@uk-augsburg.de; 3 Department of Internal Medicine II, Augsburg University Medical Center, 86156 Augsburg, Germany

**Keywords:** environmental epidemiology, cancer, mortality, climate, air quality, weather-related health, extreme events, risk assessment, statistics

## Abstract

While many authors have described the adverse health effects of poor air quality and meteorological extremes, there remain inconsistencies on a regional scale as well as uncertainty about the single and joint effects of atmospheric predictors. In this context, we investigated the short-term impacts of weather and air quality on moderate extreme cancer-related mortality events for the urban area of Augsburg, Southern Germany, during the period 2000–2017. First, single effects were uncovered by applying a case-crossover routine. The overall impact was assessed by performing a Mann–Whitney U testing scheme. We then compared the results of this procedure to extreme noncancer-related mortality events. In a second step, we found periods with contemporaneous significant predictors and carried out an in-depth analysis of these joint-effect periods. We were interested in the atmospheric processes leading to the emergence of significant conditions. Hence, we applied the Principal Component Analysis to large-scale synoptic conditions during these periods. The results demonstrate a strong linkage between high-mortality events in cancer patients and significantly above-average levels of nitrogen dioxide (NO_2_) and particulate matter (PM_2.5_) during the late winter through spring period. These were mainly linked to northerly to easterly weak airflow under stable, high-pressure conditions. Especially in winter and spring, this can result in low temperatures and a ground-level increase and the accumulation of air pollution from heating and traffic as well as eastern lateral advection of polluted air. Additionally, above-average temperatures were shown to occur on the days before mortality events from mid-summer through fall, which was also caused by high-pressure conditions with weak wind flow and intense solar radiation. Our approach can be used to analyse medical data with epidemiological as well as climatological methods while providing a more vivid representation of the underlying atmospheric processes.

## 1. Introduction

Ongoing global warming is not only manifesting itself in changes of the atmosphere and ecosystems. These changes also affect human health, e.g., by an increase in extreme events. Additionally, exposure to air pollutants, such as particulate matter and nitrogen oxides, represents an additional form of stress for organisms. The recently published 6th Assessment Report of the IPCC emphasises the extent of human influence on the global climate, declaring an increase in hot temperature extremes since 1950 as ‘virtually certain’ and describing the significant increase in greenhouse gas concentrations as ‘unequivocally caused by human activities’ [1]. In the context of climate change, various studies have also suggested adverse connections between climatic extremes and human health. Among the largest health-related threats is cancer, which the World Health Organization states as being the leading cause of premature death in over 50 countries worldwide, including Germany [2,3]. Many authors have suggested connections among meteorology, air quality, and the health of patients with an underlying cancer diagnosis [4]. Among cancer patients, the majority of deaths globally are attributed to lung cancer (18%) [5]. While there are many non-climate and -pollution related factors that lead to the onset of lung cancer, such as smoking, exposure to ambient air pollution can also have significant impacts on lung cancer incidence and mortality [6,7]. Regarding other types of cancer, several authors have pointed out similar effects, although study results vary. Turner et al. [8] suggested that there is a positive correlation between the level of PM_2.5_ and deaths from kidney and bladder cancer as well as a link between NO_2_ and colorectal cancer. They found no links between air quality and other types of cancer. In a more recent study, Turner et al. [9] could not confirm a link between bladder cancer mortality and NO_2_ or PM_2.5_. Regarding breast cancer, Tagliabue et al. [10] found an increased mortality rate under higher values of PM_2.5_. White et al. [11] confirmed these findings for a different study region, as well as finding a link with increased NO_2_ concentrations, but only for specific regions within the study area. This was, in turn, acknowledged by Hwang et al. [12], but only for incidence and not for mortality.

The findings of previous studies show an unclear picture regarding the atmospheric influence on cancer-related mortality. While studies concerning meteorological factors, e.g., temperature extremes, remain scarce, studies pointing out the adverse effects of poor air quality mostly refer to increased levels of particulate matter (predominantly PM_2.5_) and NO_2_. However, there also are findings in connection with Volatile Organic Compounds [6] and ozone [13]. These studies suggest that there are regional differences, justifying the need for further local analyses. Additionally, a distinction must be made between long- and short-term effects, which have differing effects on patients, i.e., regarding carcinogenesis and health burden [14].

This study assessed the impacts of meteorology and air quality on the mortality rate of patients with an underlying cancer diagnosis. We were also interested in the specific meteorological processes linked to the emergence of significant atmospheric states related to cancer mortality. Hence, we analysed the short-term effects by adopting a mixed epidemiological and climatological approach and linking the results to medical data. First, we assessed the overall associations between relevant predictors and cancer-related mortality by applying U tests at the seasonal level and for up to 13 lead days. Predictors included NO, NO_2_, PM_10_, PM_2.5_, and O_3_ as well as eight meteorological predictors, including temperature and humidity. This necessitated the use of a case-crossover procedure to account for the isolated effects of each predictor. In a second step, we formed composites of predictors with overlapping significant periods and performed an in-depth analysis of these periods by applying a Principal Component Analysis (PCA) to sea level pressure data. Through this, we obtained patterns related to the atmospheric circulation, improving the interpretation of the resulting meteorological states. We sought to offer an approach that could be used to detect cancer-related weather, climate and air pollution events, aiming to enhance possibilities in the field of prevention and care. This article is structured as follows: Section 2 describes the data, i.e., station-based air quality and meteorological data (Section 2.1), cancer mortality data (Section 2.2) and ERA5 reanalysis data (Section 2.3) as well as the methods, i.e., the consideration of trends and seasonality (Section 2.4), the Mann–Whitney U Test (Section 2.5), the Case-Crossover analysis (Section 2.6) and *t*-mode PCA (Section 2.7). The results are shown in Section 3, including a comparison of cancer-related extreme mortality events with non-cancer-related mortality, followed by a discussion in Section 4. Conclusions are drawn in Section 5.

## 2. Materials and Methods

### 2.1. Air Quality and Meteorology Data

Air quality data were obtained from the online archive of the Bavarian Environmental Agency (Bayerisches Landesamt für Umwelt [15]). While, at first, all provided pollutants were considered, nitric oxides (NO & NO_2_), particulate matter (PM_10_ & PM_2.5_) and ozone (O_3_) were taken into the final analysis, after having passed tests for homogeneity and completeness [16,17]. Although the analysed datasets were all classified as ‘useful’, there were a few occasions in which a break was detected for NO and NO_2_ in the year 2010. This is in accordance with the implementation of the ‘Low Emission Zone’ in Augsburg, which led to slight but significant reductions in nitric oxide concentrations [18]. As we were interested in connections with ambient air pollution and the atmospheric circulation, we used background stations within the urban area of Augsburg in order to avoid influences from heavy traffic. Primarily, the suburban background station located at the Bavarian Environmental Agency (A-LfU) was used. Due to missing values, suburban NO und NO_2_ datasets were replaced by measurements at Augsburg-Bourges-Platz (urban background). While all data are provided on an hourly basis, ozone values were aggregated to the daily maximum, and nitric oxides, and particulate matter were aggregated to the daily mean.

**Figure 1:**
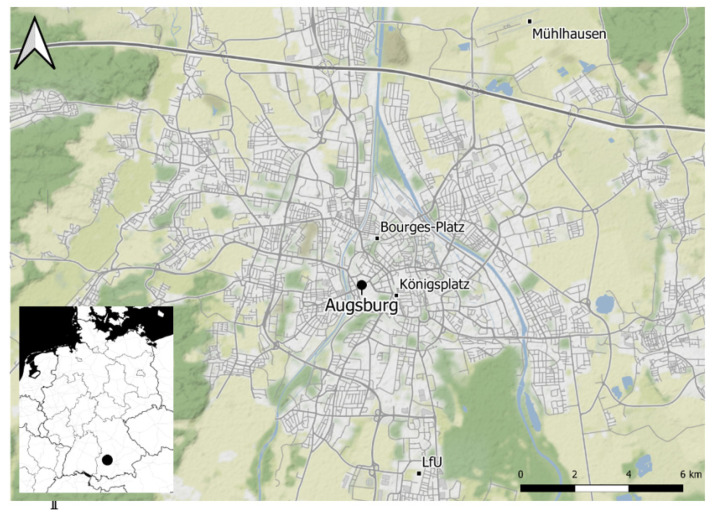
Location of meteorological and air quality measuring sites in Augsburg, Germany.

Daily meteorological data originated from the Augsburg-Mühlhausen measuring station, operated by the German Weather Service (Deutscher Wetterdienst [19]). The selected variables were the daily minimum, mean and maximum temperatures; the mean humidity; the mean and maximum wind speeds; the rainfall amount and the mean pressure. The meteorological datasets were also ‘complete’ and classified as ‘useful’. All predictors and the measuring entities are listed in Table 1, and the study region is shown in Figure 1.

### 2.2. Cancer Mortality Data

The anonymised dataset of the Bavarian Cancer Registry (Augsburg Regional Center) [20], which comprises around 40,500 deaths of patients with an underlying cancer diagnosis in the period 2000 to 2017, serves as a medical basis. The data provide information on the date of death, zip code of residence, gender and age, as well as the type of cancer. The latter includes the specification of the International Classification of Diseases code (ICD-10). We included patients who had a diagnosis assigned to ICD-10-chapter II, block C00-D48 (Neoplasms) [21]. In our study, 15,601 patients residing in the Augsburg urban area (first three zip code numbers 861) were included. For the remaining patients residing outside Augsburg, exposure to the meteorological and air-hygienic conditions in Augsburg could not be assumed. The analysis was carried out for the period 2000–2017. As stated by Grundmann et al. [22], for Augsburg, there has been a recent decline in incidence for some cancer entities. Still, cancer remains a predominant factor in mortality, with 29% of all diseased individuals in 2017 having had a documented cancer diagnosis. We also found this trend in our analysis. In order to compare cancer mortality with the total mortality in the city of Augsburg, mortality data from the Bavarian State Office for Statistics [23] were also included.

Regarding the medical relevance and possible preventive measures when considering the mortality of cancer patients, days with a significantly increased number of deaths were of particular interest. The 95th percentile proved to be suitable for defining these cancer-related high mortality events. In this case, enough days were included in the analysis so that the effect of an outlier did not distort the statistical characteristics, while at the same time, the events remained within the range of extremes. Our data showed an annual cycle of daily death totals, with higher total sums in winter. To avoid the influence of seasonality on the selection of high-mortality events, the 31-day moving 95th percentile was computed for each calendar day and defined as the threshold. Specifically, the corresponding 95th percentile had to be exceeded to recognise a specific day as a high-mortality event. Thus, in summer, an event was defined as a daily sum of deaths of 6 or higher; in winter, this was 7 deaths or higher. Out of the 6575 days analysed between 2000 and 2017, 222 days exceeded the corresponding threshold and were considered cancer-related high-mortality events.

### 2.3. SLP Reanalysis Data

For the computation of the *t*-mode PCA, gridded daily mean sea level pressure data were obtained from the ERA5 reanalysis for the period 2000–2017, provided by the European Centre for Medium-Range Weather Forecasts (ECMWF, [24]). Computations were performed for a 1° × 1° grid in the 25° W–40° E and 25° N–70° N domain. Augsburg is located near the center of the domain at 48.3° N, 10.9° E.

### 2.4. Trends and Seasonality

To account for trends and seasonality, each meteorological and air-quality-related variable firstly underwent a detrending process. A variety of methods for detrending exist [25,26,27]. We followed an approach in accordance with Iler et al. [28] and applied a simple linear regression to each variable. The original data were then subtracted from the regression values; hence the residuals underwent further analysis as detrended values. In order to account for seasonality, for each calendar day, a 31-day moving average was computed and then subtracted from the corresponding detrended data. We, therefore, considered detrended, season-adjusted daily anomalies of atmospheric predictors in our study. Under this setup, negative (positive) values of predictors described a below-average (above-average) state regarding the corresponding moving average of the specific day. This approach has also been used in former studies [29,30,31].

### 2.5. Mann–Whitney U Test and Predictor Correlations

**Figure 2:**
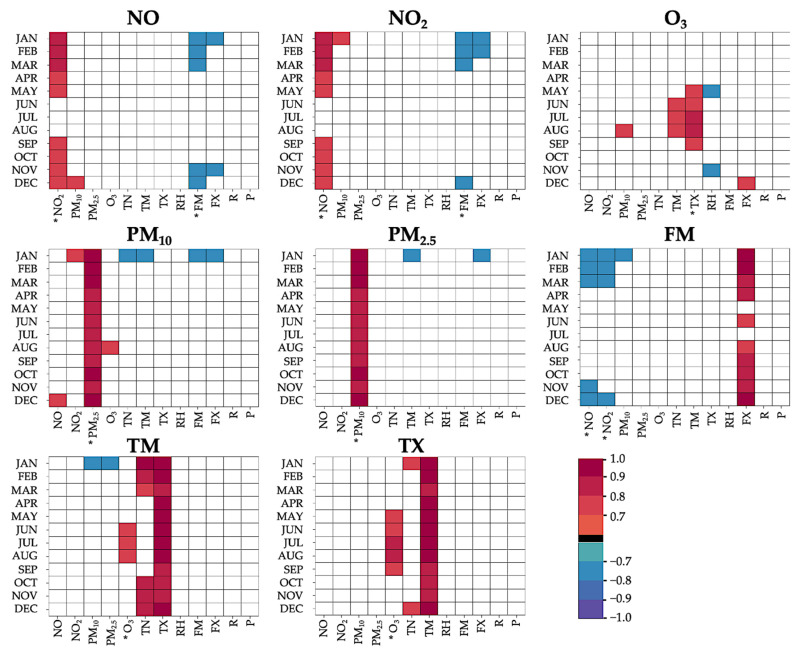
Monthly predictor correlation coefficients for all predictors with respect to the case-crossover analysis. Only coefficients >0.7 and <−0.7 (=shared variance approx. 50%) were plotted. Variables for which case-control was necessary are marked with *.

To find significant anomalies in meteorological and air quality data when comparing high with average mortality events, a Mann–Whitney U test [32,33] was carried out, as the predictor variables did not follow a normal distribution. Analyses were done for 3-month seasons. The mortality event days as well as up to 13 lead days were used. Lead days were not considered singularly, but comprised all days in a specific lead period, including the event day. We sought to find the unique influence of each variable on high-mortality days. In this regard, the necessity of a case-crossover design was evaluated by Spearman correlation coefficients between the various predictor variables (Figure 2).

Predictors of non-similar types (e.g., temperature-related, and ozone-related) with an absolute correlation coefficient higher than 0.7 (i.e., 50% shared variance) for three or more consecutive months were subject to a case-crossover analysis, while non-correlated predictors underwent U testing directly. Note that if more than one variable of a similar type required case-crossover analysis (e.g., TM and TX regarding ozone), only one variable of the specific type was regarded, according to the largest range of months with |r| > 0.7 and the absolute sum of explained variance. Case-crossover analysis was applied on a single-monthly basis if at least one month in a 3-month-moving window complied with the requirements for case-crossover. Afterwards, results were aggregated back to a three-monthly window.

### 2.6. Case-Crossover

Case-crossover analysis, as suggested by Pinheiro et al. [34], aims to uncover unique effects of predictors by controlling a specific variable by its high-correlated, confounding variables. By applying this procedure, the values of a specific variable on high-mortality days (=case) were tested against values on average mortality days (=control) by matching case days to corresponding control days with the highest similarity in the correlated variables. In our study, we developed a form of case-crossover analysis that was optimally adapted for our data in accordance with Pinheiro et al. with the following conditions: control days had to be dated within the same month, although we did not distinguish between different days of the week. This was due to the lack of control days when applying the case-crossover analysis to lead periods. In our case, a control value was determined by the least error sum of the correlated variables for the purpose of integrating lead periods. Hence, we integrated control periods instead of single control days. The deviation of all case and control values within a 14-day period was weighted and summed for every control period candidate. Weighting was applied using a half-normal distribution so that the error near a high-mortality event was given greater importance than deviations of more remote days. This procedure also allowed for the consideration of more than one control variable. All case periods and the corresponding control periods with the lowest overall weighted error sum for their preceding period subsequently underwent U testing. Variables requiring case-crossover procedures, the corresponding control variables and the months in which case-crossover was necessary are shown in Figure 2.

### 2.7. Principal Component Analysis (PCA)

For a deeper climatological interpretation of relevant months and lead periods before high-mortality events, the underlying atmospheric circulation, resp. weather types, was considered. This provided information about the general temperature and humidity conditions and the accumulation of airborne substances. In addition, the lateral transport of airborne substances can be explained by the resulting atmospheric flow during a specific weather situation.

While U testing was used to provide overall information on significant months and lead periods regarding high-mortality events on a single-predictor basis, we further characterised these specific time periods by applying PCA to the sea level pressure data from the ERA5 reanalysis dataset. Before the application of the PCA, we reduced the complexity of weather situations by combining contemporaneous predictors, i.e., they underwent processing in order to find the optimum months and lead period. Firstly, we defined a maximum time range by using the maximum outer extent of monthly windows and lead periods, in which every included predictor of interest showed significant U test results. Secondly, for every possible option within the maximum extent (m lead periods x n months) the quotient of the reduction in size compared with the maximum extent and the ratio of time steps in which not every predictor was significant was computed. The optimum time range was determined by the combination of m and n with the highest quotient value. For this optimum time range, a *t*-mode rotated PCA (VARIMAX) (e.g., Philipp [35], Huth [36]) was applied. Thus, as input, the sequence of days from the identified optimum lead time up to the mortality event day were entered into the PCA, as output weather type sequences typical for mortality events were generated.

## 3. Results

### 3.1. Cancer-Related High-Mortality Events

Deaths within cancer-related mortality events were found to be distributed unequally among age groups, with about 55% in patients aged 75 years or older (higher female rate than male), about 37% in the 55–74 age group (higher male rate), and just under 8% in the 25–54 age group (balanced rates). The most common underlying cancer was lung cancer (ICD C34, 12%). This was followed by breast cancer (ICD C50, 10%), colorectal cancer (ICD C18, 8.5%) and prostate cancer (ICD C61, 8%). With respect to the annual distribution of mortality events, a high level of similarity between summer (110 events) and winter (112 events) was found due to the choice of a moving percentile. In this context, it must be noted that the original total sum of deaths was higher in winter (780) than in summer (705). Additionally, looking at the monthly sum, months with an increased number of deaths in cancer-related mortality events were detected (January, April, July, October) next to months with a low total sum of deaths on event days (February, June, September, December).

Across all high-mortality events, there were minimal within-group differences in the characteristics of age groups, genders, and cancer types. Although it must be noted that, while most diagnosis groups showed higher total sums of deaths on event days in winter, a reverse effect was found to occur for patients with breast cancer, resulting in a higher total sum of daily deaths in summer. This surplus was greatest in July and August. Cancer-related mortality events did not occur in clusters and can, therefore, be understood as individual peaks. During winter, events were followed by other events on the following day only four times throughout the considered 18-year period. In summer, this only occurred three times. Spells longer than two days did not occur.

### 3.2. Relations to Weather and Air Quality

**Figure 3:**
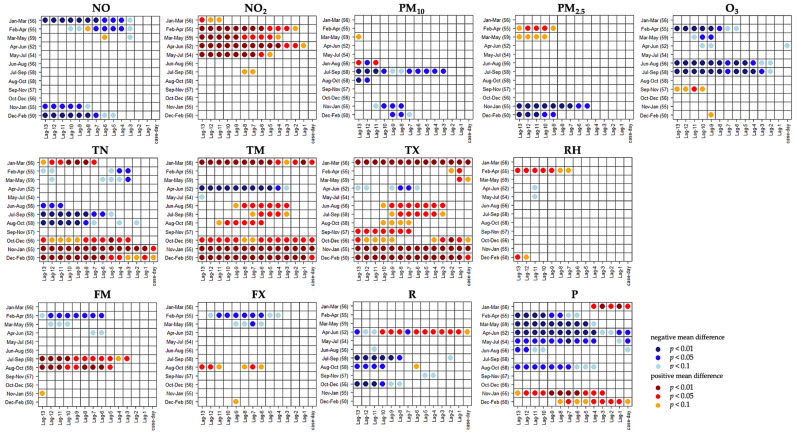
U test results for meteorological and air quality predictors regarding cancer-related mortality events. Monthly windows and up to 13 days prior to each event were considered. Variable abbreviations are listed in Table 1. Numbers next to monthly windows indicate the sum of mortality events within given months. Coloring refers to the mean difference of event-related days minus non-event-related days. Red indicates a significant positive deviation for high-mortality events, while blue indicates a significant negative deviation. *P*-values of significance are provided for three levels (0.01, 0.05, 0.1).

For various atmospheric parameters, significant anomalies were revealed in the U test in the run-up to cancer-related mortality events (Figure 3). Regarding air pollutants, NO_2_ and PM_2.5_ appear to be particularly relevant. In the case of meteorological variables, temperature anomalies, in particular, were prominent. Air pressure also showed significant manifestations in many cases, although these were primarily relevant in climatological interpretation. Significantly higher NO_2_ concentrations occurred in February through July, approximately 4 days to two weeks before mortality events. Elevated PM_2.5_ concentrations were significant for February through May between 10 and 13 days in advance of mortality events. In the case of temperature, various significant relationships emerged. Between October and March, higher than usual temperatures occurred throughout the two weeks prior to corresponding high-mortality events. Between February and May, cool minimum temperatures were often significantly prominent in the medium- to long-term lead-up. During summer and fall (June through October), a combination of significantly warmer maximum and cooler minimum temperatures was observed.

Temporally overlapping significant anomalies of multiple variables were combined in the form of composites. Increased concentrations of NO_2_ and PM_2.5_ occurred between February and April, together with lower than usual minimum temperatures, 12–13 days before a mortality event. This effect also occurred 4–5 days before an event when considering February through May, as well as a combined effect of NO_2_ and PM_2.5_ 10–13 days before an event. In the medium-term lead period (6–9 days), elevated NO_2_ concentrations occurred between April and June in association with cooler mean and maximum temperatures. Significantly above-average mean and maximum temperatures occurred in summer and early fall (June through September) 3–8 days before an event, coinciding with below-average low temperatures during a more extended lead period (7–13 days). O_3_ also exhibited a coupled effect with maximum temperature 10–13 days before corresponding mortality events in fall. Subsequently, *t*-mode PCA was applied to the detected composites. The composites are recorded in Table 2.

First, a single high-mortality event within a specified time range was assigned to its matching principal component (PC) by the highest absolute correlation coefficient (loading) to the scores obtained from PCA. Within a selected PC, the atmospheric states in advance of each assigned event could be demonstrated and explained by the atmospheric circulation (scores) proposed by PCA. Means of relevant variables are given for corresponding lead days assigned to a similar PC. Means in the following text only represent cases in which an event had positive loading towards the illustrated atmospheric state, as cases with negative loadings were neglected due to low occurrence (1 in 52). It is important to note that different or contradictory results regarding U testing might occur when analysing event-based variables, as U test samples contain every event, while score-allocated samples represent a subset. The overall findings of the PCA outputs are addressed in the discussion section. In the following text, we demonstrate the capabilities of this approach by exemplifying it for a specific composite. We selected the fourth composite (Table 2), for which U testing suggested above average NO_2_ concentrations as well as below average mean and maximum temperatures in April through June.

### 3.3. PCA Results

PCA applied to composite number 4 suggests eleven PCs, with an overall explained variance of 82%. We take a closer look at the first, second, fourth and fifth PC. The third PC comprises a strong westerly flow over the target region resulting in low pollutant concentrations and was therefore left out in the detailed analysis. PCs one and two inherit the highest frequency and largest values of explained variance, PCs four and five are less frequent but inherit notable conditions of high interest. Composite number 4 shows significant impacts of predictors for the days 9 to 6 in advance of a high mortality event during April through June. The consecutive period of the ninth to sixth day before every high mortality event during April through June is treated as a unit and handed to PCA. Under this setup, the application of PCA makes it possible to investigate typical sequences of weather patterns that occur during the given consecutive days in advance of high mortality events. The resulting PCs each represent one of the typical sequences of weather patterns, that is followed by a high mortality event. In the case of composite number 4, the high mortality event occurs 6 days after the last day of the given sequences.

#### 3.3.1. PC 1

**Figure 4:**
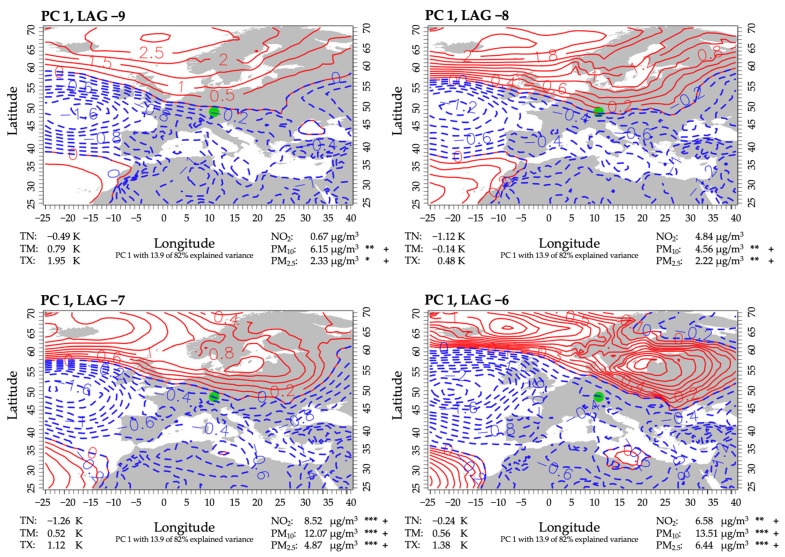
Weather sequence of PC 1 for lag days 9 to 6 before a high mortality event in April through June. Contours are scores of PC 1 and refer to the sea level pressure on a given day, with red indicating high pressure and blue indicating low pressure. Also given are mean predictor anomalies of all corresponding days of the same lag for air temperature and the chosen air pollutants (for abbreviations, see Table 1). Positive values indicate an above-average state, and negative values indicate a below-average state. If the values of a specific lag day deviate significantly from all non-high-mortality-related days within April through June, the level of significance is provided (***: 0.01; **: 0.05; *: 0.1; determined by U test) as well as the mean difference of all days of the same lag minus the mean of all non-high-mortality-related days, indicated by + or −. + indicates a higher anomaly mean on the corresponding lag days than on non-high-mortality-related days.

PC 1 accounted for 13.9% of the explained variance and was assigned to 19% of the 52 events. Under the given atmospheric state, Augsburg was under the influence of a distinct high-pressure system over Scandinavia and Eastern Europe, intensifying throughout the considered sequence (Figure 4). The resulting conditions comprised a persistent easterly flow throughout the entire lead period, connected with clear conditions and the advection of warm and dry continental air masses. However, especially in spring (April, May), clear skies resulted in strong nocturnal cooling, causing low minimum temperatures. These thermal effects were shown by an increased daily maximum temperature by up to 1.9 K, near-average mean temperatures as well as mostly below-average minimum temperatures. Within a period of three days mean NO_2_ concentrations increased from below-average to about 7.5–8.5 µg/m^3^ above normal. This increase led to significantly higher concentrations compared with non-event-related days during the April to June period. Comparable increases toward significantly higher concentrations were also detected for PM_10_ and PM_2.5_. Causes for this could be increased emissions from heating, especially during cold nights, as well as air pollution accumulation in the lower atmosphere due to low-flow inversion-type conditions. Regarding high PM-concentrations, lateral transportation from Eastern Europe must also be considered.

#### 3.3.2. PC 2

**Figure 5:**
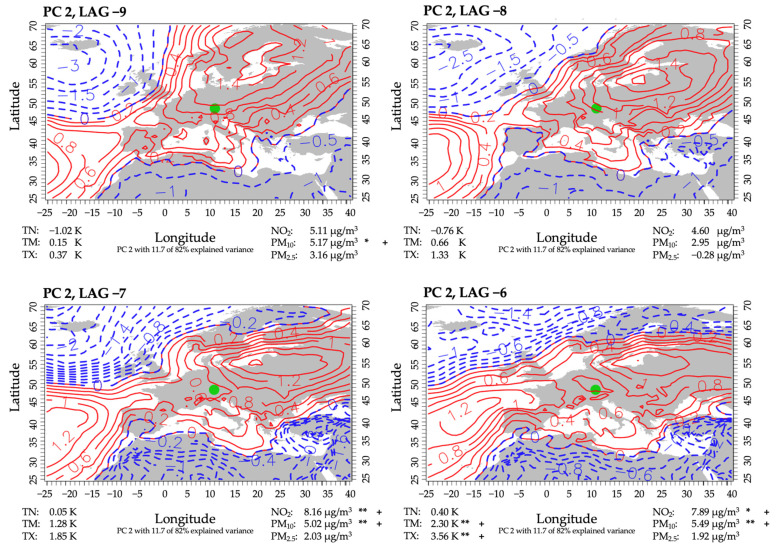
Same as Figure 4, but for PC 2. The level of significance is provided (**: 0.05; *: 0.1). The mean difference of all days of the same lag minus the mean of all non-high-mortality-related days, indicated by + or −. + indicates a higher anomaly mean on the corresponding lag days than on non-high-mortality-related days. − indicates a lower anomaly mean on the corresponding lag days than on non-high-mortality-related days.

Nineteen percent of all events that occurred in April through June were assigned to this PC, accounting for an explained variance of 11.7%. Although there were similarities to PC 1 concerning the atmospheric circulation, PC 2 showed a pronounced high-pressure system shifted to the east and reaching further into Central Europe (Figure 5). Therefore, Augsburg was affected by weak easterly flows of dry-continental airmasses and clear skies. Again, the effect of nocturnal cooling was expressed by below-average minimum temperatures. The plotted weather sequence showed a continuing persistence of this weather type, leading to steadily increasing mean and maximum temperatures, reaching significantly higher than usual levels. Throughout the four-day period, concentrations of air pollutants were mostly above average, reaching significance for NO_2_ and PM_10_ six days before a high-mortality event.

#### 3.3.3. PC 4

**Figure 6:**
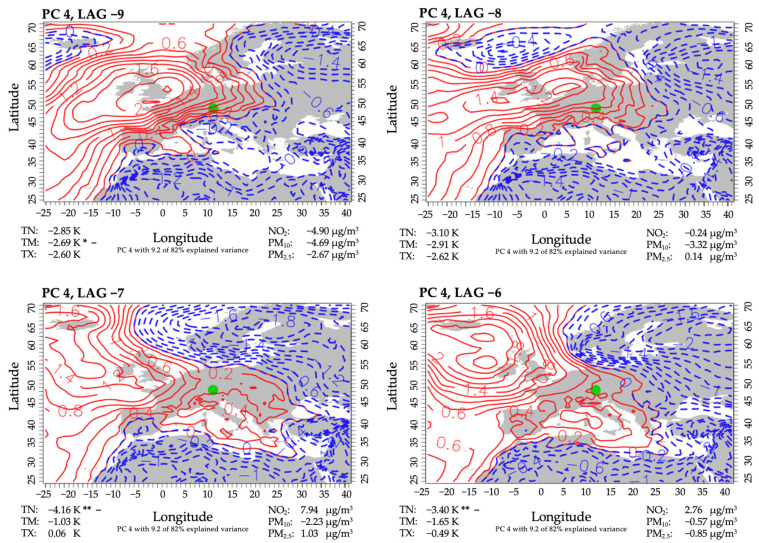
Same as Figure 4, but for PC 4. The level of significance is provided (**: 0.05; *: 0.1). The mean difference of all days of the same lag minus the mean of all non-high-mortality-related days, indicated by + or −. + indicates a higher anomaly mean on the corresponding lag days than on non-high-mortality-related days. − indicates a lower anomaly mean on the corresponding lag days than on non-high-mortality-related days.

Three out of 52 events were assigned to the fourth PC, which accounted for 9.2% of the explained variance. In this case, a high-pressure system was dominant over the British Isles (Figure 6). Throughout the period, the high-pressure ridge reached out to the Mediterranean, thus also affecting Central Europe. At first, flows in Augsburg originated from the eastern direction. The change in the position of the high-pressure system as well as the persistent extension of the ridge caused the wind direction to shift north. Advection of polar airmasses resulted in below-average temperatures, as expected. Although PM concentrations were mostly lower as usual, a marked increase throughout the period was observed. NO-concentrations started at below-average values as well and experienced a mean relative increase of almost 13 µg/m^3^ within two days.

#### 3.3.4. PC 5

**Figure 7:**
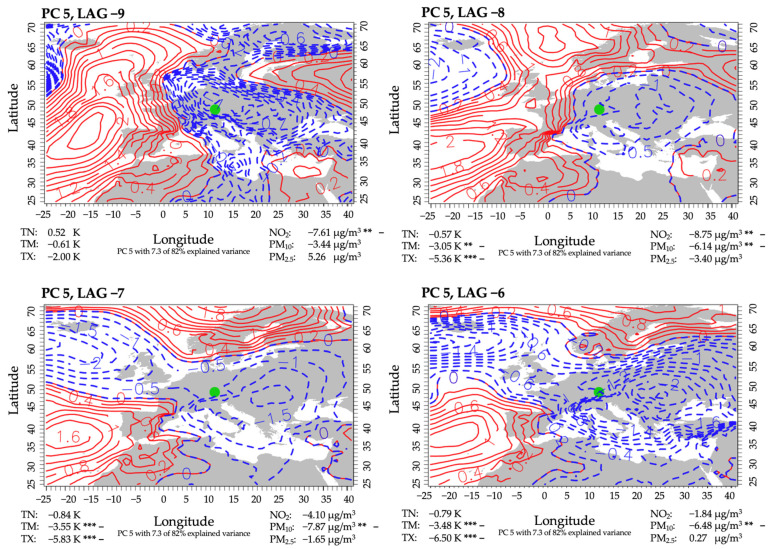
Same as Figure 4, but for PC 5. The level of significance is provided (***: 0.01; **: 0.05). The mean difference of all days of the same lag minus the mean of all non-high-mortality-related days, indicated by + or −. + indicates a higher anomaly mean on the corresponding lag days than on non-high-mortality-related days. − indicates a lower anomaly mean on the corresponding lag days than on non-high-mortality-related days.

PC 5 accounted for 7.3% of the explained variance and included six mortality events in cancer patients. Throughout the period, Augsburg was persistently under the influence of a low-pressure system, which slowly shifted its center from Central to Eastern Europe (Figure 7). During the period, the low-pressure system varied in intensity. Resulting flows in Augsburg, therefore, shifted from northwest to north and, eventually, northeast, transporting cool airmasses in combination with windy and rainy conditions under the influence of a low pressure. This resulted in below-average temperatures. In cases of mean and maximum temperatures, they were several K below usual, and this effect was highly significant when compared with non-event-related days in the same period. While mean anomalies were mostly significantly lower than usual for NO_2_ and PM_10_, PM_2.5_ experienced a slight increase towards the end of the period. In addition, it must be noted that NO_2_-concentrations, while remaining below average, increased similarly to the other inspected PCs, in this case by about 7 µg/m^3^ within two days.

### 3.4. Relationship of Cancer-Related Mortality Events to Non-Cancer-Related Mortality

For the comparison with the total mortality in Augsburg, the daily sum of cancer-related deaths was first subtracted from the total mortality rate. This concerned 29.6% of all deceased patients, as also been stated by Grundmann et al. [22]. After applying the Spearman correlation analysis, no statistical relationship was found between daily cancer-related deaths in Augsburg and the remaining daily mortality (r_s_ ≈ −0.07, *p* ≈ 0). We then conducted an analysis of moderate extreme events for the remaining daily mortality by applying the procedure described in Section 2.5 and Section 2.6. According to our definition, a non-cancer-related extreme event was recorded if the daily sum exceeded 10–11 cases, depending on the respective day in summer, and 11–12 cases in winter. Temporal coincidence of the 95th percentile-based high mortality events in cancer patients and non-cancer-related mortality events showed no significant overlaps. However, U testing and case-crossover analysis, under the identical setup previously described, showed interesting results regarding similarities and differences to cancer-related events.

The U test significance and the indication of the overall mean difference between non-cancer related event days and non-event days, analogous to Figure 3, are provided in the Supplementary Materials Figure S1. Several variables with a high relevance for cancer also appeared to be significant for non-cancer-related extreme events, including NO_2_, PM_2.5_ and the minimum temperature. For the former two, this included significantly above-average concentrations in late summer through winter. Minimum temperatures deviated significantly to negative values in late winter and appeared above-average in summer and fall. However, although a similarity in the overall importance of these variables for cancer- and non-cancer-related mortality events was detected, it became clear that there were notable variations in the direction of the effect (positive or negative) as well as with respect to the relevant months and lead times of these predictors. Other predictors with no significant effects on cancer-related mortality appeared to be significant for non-cancer-related mortality. This, for example, included significantly above-average O_3_ concentrations in summer and fall as well as significantly below-average relative humidity, coupled with overall below-average temperatures in winter through early spring.

## 4. Discussion

The discussion of our results can be divided into two sections. Firstly, the relationship between atmospheric states and the occurrence of cancer-related moderate high mortality events is covered. Secondly, we detail the information gained through the application of climatological methods to medical data.

While the assessment of long-term effects remains a major part of epidemiological research, we aimed to present the short-term effects of meteorology and air quality on cancer-related mortality in Augsburg, Southern Germany during the 2000–2017 period. We focused on moderate extreme events regarding mortality by defining the 31-day-moving 95th percentile as the threshold for cancer-related mortality events. The medical evidence states that carcinogenesis is mostly affected by long-term factors, i.e., persistent exposure to pollutants, such as particulate matter [14,37,38,39]. Short-term effects place an additional burden on cancer patients. An organism that is additionally burdened, for example, by increased pollutant concentrations or significantly low or high temperatures, has greater susceptibility to the onset of additional diseases, mainly of a respiratory nature [40,41], which, in turn, promote premature death. Our study suggests that above-average concentrations of NO_2_ and PM_2.5_ as well as temperature-related effects contribute the most regarding these burdens for individuals in Augsburg, predominantly occurring one to two weeks before death events.

NO_2_ showed significantly above-average anomalies, beginning in February and lasting until mid-summer. These anomalies mostly occurred 4 to 13 days before a mortality event. Higher than usual PM_2.5_ concentrations occurred from February to May, preceding high-mortality events by 10 to 13 days. Above-average concentrations during this period often occurred in combination with lower than usual temperatures. From summer through autumn, significantly above-average maximum and mean temperatures occurred in combination with below-average minimum temperatures in advance of mortality events. Our analysis indicates that lag effects for warmer temperatures are shorter (3–8 days) when compared with low temperatures (7–13 days). Another season with significantly above-average temperatures was shown to be winter, from November through February.

The finding that there is an overall higher risk of death under extreme weather conditions and bad air quality is in accordance with other studies [42,43,44,45,46]. A strong link to respiratory mortality can be assumed. Many authors have indicated that there is a strong link between respiratory mortality and short-term exposure to increased levels of ambient PM_2.5_ and SO_2_ [47,48,49]. While SO_2_ was excluded from our analysis, our study confirms the major role of PM_2.5_. Recent studies have also indicated the adverse respiratory health effects of increased NO_2_ levels [50,51]. A study distinguishing between respiratory-related and cancer-related deaths found a distinct increase in mortality risk among lung-cancer patients when exposed to high levels of ambient NO_2_ [52]. A recently published meta-analysis showed strong evidence of adverse effects of high PM_2.5_ and NO_2_ concentrations, among other pollutants, and a resulting increase in cancer-related mortality [4]. The authors also pointed out the limitations of these studies, especially the focus on non-lung-related cancer types, and the need for further research. Although our data suggest a strong link between PM_10_ and PM_2.5_, the isolated analysis of both variables in our case showed notable, significant anomalies only for the latter. The PCA analysis, in turn, revealed significantly high levels of PM_10_, in connection with favorable synoptic conditions.

In the context of temperature, our findings were mostly in accordance with previous studies. While specifically cancer-related analyses regarding temperature extremes are scarce, an increase in overall mortality with significantly low or high temperatures has been observed [53]. While elderly patients form the largest group regarding cancer-mortality, it must be noted that, in this context, elderly patients are generally more likely to be affected by significant temperature anomalies.

An increase in air pollution concentrations can stem from three major causes: in situ formation, accumulation and lateral transport. Favourable conditions for the accumulation of air pollutants include low wind speeds and no washout by precipitation [54,55]. These conditions are best-matched with anticyclonic and low-flow patterns in general and become particularly relevant in winter when temperatures are low and emissions from traffic and heating are high. Lateral transport of airborne substances, in general, can lead to an increase in concentrations, in particular when originating from specific locations such as traffic- or industry-intense and highly populated urban areas. For our study site, specific conditions apply. As a study conducted in Germany [56] pointed out, lateral transport from western European locations plays only a minor role, whereas flows originating from Eastern Europe become dominant in this context. In combination with high pollutant concentrations over Southern and Eastern Europe [57,58], lateral transport under weak easterly to southerly flows and dry conditions must be considered when analysing bad air quality at our study site. For composites 1–4, above-average NO_2_ and particulate matter concentrations as well as below-average temperatures were detected. The PCA suggested that these conditions are caused by (a) anticyclonic conditions with southerly to easterly flows and (b) low-flow conditions. Warm temperature anomalies in summer and early fall (composites 5–7) were accompanied by low levels of cloud coverage, allowing for intense radiation, as well as subtropical flows with southern origins. Nocturnal cooling increased in the further course of late summer to fall, resulting in below-average minimum temperatures. Significantly above-average temperatures from November through February can be explained by a dominant impact of westerly flows, bringing Augsburg under the influence of warmer maritime air masses. This is contradictory to the other results, as positive temperature anomalies in winter in our domain refer to overall mild conditions, bearing no adverse health effects. However, PCs 3 and 4 of this period, which had an explained variance of 22%, described a strong anticyclonic influence, resulting in easterly flows, leading to deviations of up to 3.3 K below average for minimum and 4.5 K below average for maximum temperatures. This, in turn, is in accordance with the results of previous studies concerning the health effects of low temperature extremes.

Although a significant contribution of atmospheric predictors can be established, it must, nevertheless, be noted that other, non-atmospheric factors contribute to cancer mortality as well [59,60,61]. In our case, we focused solely on the effects of atmospheric predictors on extreme cancer-related mortality events. To account for non-atmospheric factors, more detailed information about the patients would be necessary, e.g., cause of death, lifestyle parameters, place of residence and pre-existing conditions. Further advancements could also be achieved by incorporating more cases in a cross-regional analysis.

Predominant predictors regarding cancer-related mortality can also be of high relevance for non-cancer-related mortality. This is in accordance with the former studies discussed above and comprises NO_2_, PM_2.5_ and deviations in temperature. However, there appear to be significant differences in the seasonal and temporal (lead time) characteristics of these predictors as well as in the overall direction of the effects. The latter is, for example, expressed by below-average temperatures for non-cancer-related mortality events from January through March, while the opposite is the case for cancer-related events. Adverse effects of low temperatures on overall mortality were, for example, pointed out by Gasparrini et al. [43]. Additionally, ozone appears to be of high importance for non-cancer mortality, which is supported by the findings of Hertig et al. [62,63]. Still, these findings must be interpreted cautiously, as patient information in the overall mortality dataset is limited. Due to this, we could not distinguish influential pre-existing conditions and the cause of death. Further analysis could be useful for providing a deeper understanding of these links in future studies.

Regarding methods, our study shows the potential of statistical hypothesis testing as an alternative to the use of statistical models. This applies, in particular, when modeling is aggravated, e.g., due to a low number of cases. U testing can provide a broad overview of links between medical data and atmospheric predictors. Its strengths lie in the analysis of seasonality and lag-periods. While overall responses become clear, the specific meteorological conditions in the period before such mortality events remain unknown. We, therefore, applied PCA to the results of predictor-based U testing and highlighted the range of meteorological conditions linked with the corresponding period. This approach clearly demonstrated that specific meteorological and air pollution anomalies need not stem from the same weather types. An in-depth analysis of weather types, in particular weather sequences, allowed for a robust interpretation of the relevant anomalies.

## 5. Conclusions

In our study, we found significant deviations in meteorological and air quality variables in the run-up to cancer-related mortality events. We found the main drivers to be elevated NO_2_ and PM_2.5_ concentrations in the late winter to early summer period, together with below-average minimum temperatures. In summer and fall, cancer-related mortality events were linked with significantly higher mean and maximum air temperatures. More than half of the decedents considered were 75 years or older, and more than 90% were older than 54. The total number of decedents was higher in winter than in summer. An exception was the group of breast cancer patients, for whom increased mortality occurred in summer. The combination of pathophysiological and climatological views on the present results suggests that the influences of air quality and weather do not have direct short-term effects on carcinogenesis, but rather, represent an additional burden on cancer patients. We also inspected the impacts of the meteorological and air quality variables on overall mortality. Although predominant predictors for cancer-related mortality are also of high importance for non-cancer-related mortality, noticeable differences regarding the characteristics of the relationships were shown. The results show the potential for further investigations of the relationship between cancer mortality and atmospheric conditions. A detailed knowledge base about the atmospheric conditions in the run-up to cancer-related mortality events could help to identify and predict adverse atmospheric conditions in advance of their occurrence and, thus, could be used to enhance the preparedness and response within health care.

## Figures and Tables

**Table 1:** Included atmospheric predictors, corresponding measuring sites and measuring entities.

Abb.	Variable	Unit	Measuring Site	Measuring Entity
NO	nitric oxide	µg/m^3^	A-Bourges-Platz	LfU Bayern
NO_2_	nitrogen dioxide	µg/m^3^	A-Bourges-Platz	LfU Bayern
PM_10_	particulate matter	µg/m^3^	A-LfU	LfU Bayern
PM_2.5_	particulate matter	µg/m^3^	A-LfU	LfU Bayern
O_3_	ozone	µg/m^3^	A-LfU	LfU Bayern
TN	minimum temperature	°C	A-Mühlhausen	DWD
TM	mean temperature	°C	A-Mühlhausen	DWD
TX	maximum temperature	°C	A-Mühlhausen	DWD
RH	relative humidity	%	A-Mühlhausen	DWD
FM	mean wind speed	ms^−1^	A-Mühlhausen	DWD
FX	maximum wind speed	ms^−1^	A-Mühlhausen	DWD
R	rainfall amount	mm	A-Mühlhausen	DWD
P	Mean sea level pressure	hPa	A-Mühlhausen	DWD

**Table 2:** Composites of contemporaneous significant predictors in advance of high-mortality events, as suggested by U testing. Only health-relevant predictors of airborne substances and temperature are shown.

Composite	Months	Lead-In Days	Above Average	Below Average
1	February–April	12–13	NO_2_, PM_2.5_	TN
2	February–May	4–5	NO_2_	TN
3	February–May	10–13	NO_2_, PM_2.5_	
4	April–June	6–9	NO_2_	TM, TX
5	June–September	3–8	TM, TX	
6	August–October	7–10	TM, TX	TN
7	September–November	10–13	O_3_, TX	
8	November–February	3–8	TN, TM, TX	

## Data Availability

Publicly available datasets were analyzed in this study. This includes (a) station-based meteorological data, (b) air quality data and (c) reanalysis data. This data can be found here: (a) https://opendata.dwd.de/climate_environment/CDC/observations_germany/climate/daily/kl/historical/tageswerte_KL_00232_19470101_20201231_hist.zip; (b) https://www.lfu.bayern.de/luft/immissionsmessungen/messwertarchiv/index.htm; (c) https://cds.climate.copernicus.eu/cdsapp#!/dataset/reanalysis-era5-single-levels?tab=overview. Restrictions apply to the availability of (a) cancer-related mortality data and (b) overall mortality. (a) was obtained from the Bavarian Cancer Registry and is available at https://www.lgl.bayern.de/gesundheit/krebsregister/auswertung_forschung/index.htm with the permission of the Bavarian Cancer Registry; (b) was obtained from the Bavarian State Office for Statistics and is available at https://www.statistik.bayern.de/statistik/gebiet_bevoelkerung/bevoelkerungsbewegung/index.html#link_2 with permission of the Bavarian State Office for Statistics (all accessed on 10 October 2021).

## Supplementary Materials

The following is available online at https://www.mdpi.com/article/10.3390/ijerph182211737/s1, Figure S1: U test results of meteorological and air quality predictors regarding noncancer-related mortality events.
