# Supplementary material for: Associations between Weather, Air Quality and Moderate Extreme Cancer-Related Mortality Events in Augsburg, Southern Germany
Source: Int J Environ Res Public Health. 2021 Nov 9;18(22):11737. doi: 10.3390/ijerph182211737 (PMC8617977; doi:10.3390/ijerph182211737)
Supplement: Supplementary file 1 [file ijerph-18-11737-s001.zip › ijerph-1437321-supplementary.pdf]

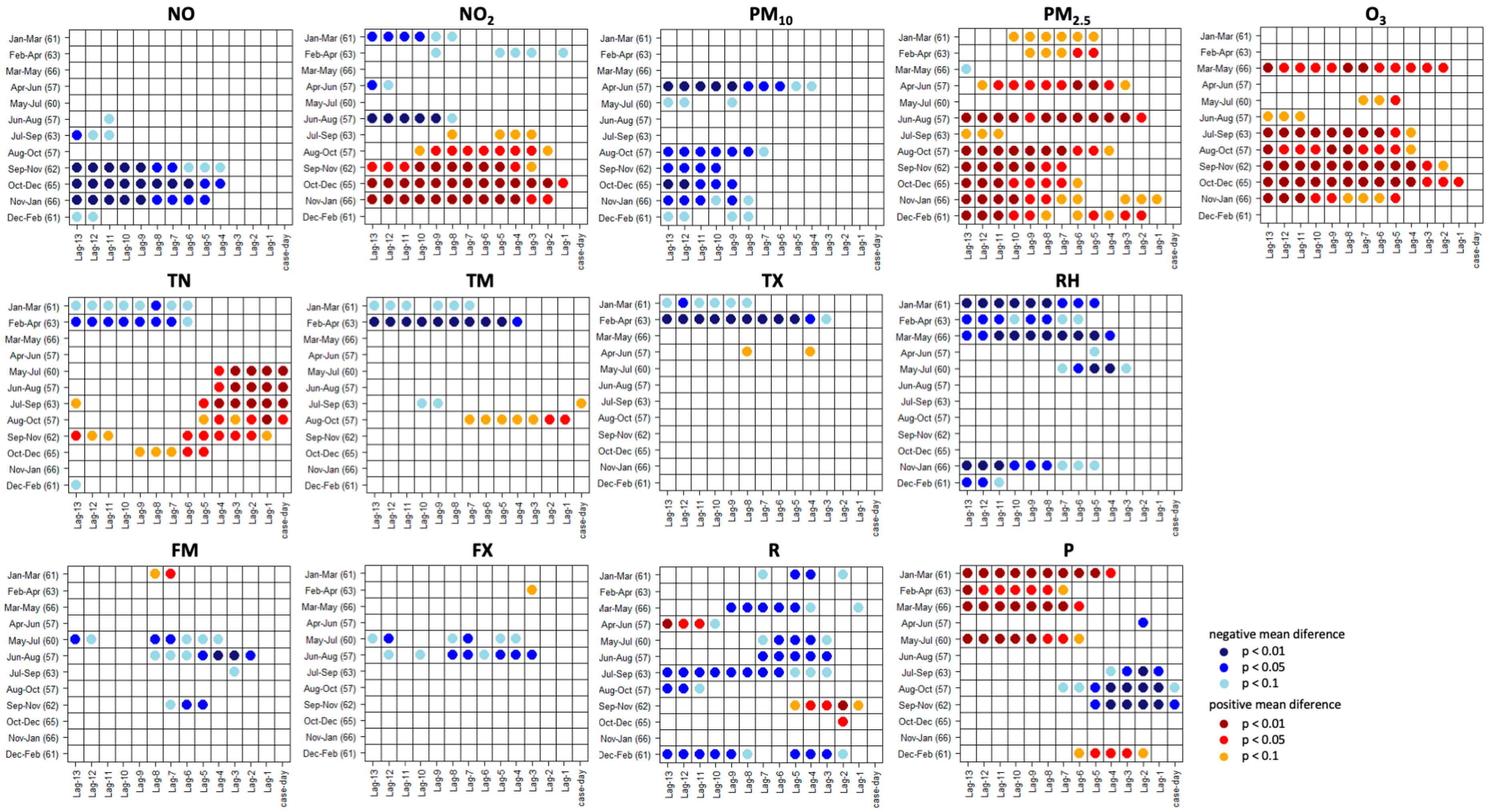

Figure S1. U test results of meteorological and air quality predictors regarding non-cancer-related mortality events. Monthly windows and up to 13 days prior to event are considered. Variable abbreviations are listed in Tab. 1. Numbers next to monthly windows indicate sum of mortality events within given months. Coloring refers to the mean difference of event-related days minus non-event-related days. Hence, red color indicates a significant positive deviation on high mortality events, blue a significant negative deviation. P-values of significance are provided for three levels (0.01, 0.05, 0.1).
